# Third party EPID with IGRT capability retrofitted onto an existing medical linear accelerator

**DOI:** 10.2349/biij.5.3.25

**Published:** 2009-07-01

**Authors:** DO Odero, DS Shimm

**Affiliations:** 1 Radiation Therapy, Raleigh Regional Cancer Center, Beckley, West Virginia, United States; 2 Radiologic Technology, Mountain State University, Beckley, West Virginia United States

**Keywords:** Portal Imaging, Simulation, EPID, IMRT, IGRT

## Abstract

Radiation therapy requires precision to avoid unintended irradiation of normal organs. Electronic Portal Imaging Devices (EPIDs), can help with precise patient positioning for accurate treatment. EPIDs are now bundled with new linear accelerators, or they can be purchased from the Linac manufacturer for retrofit. Retrofitting a third party EPID to a linear accelerator can pose challenges. The authors describe a relatively inexpensive third party CCD camera-based EPID manufactured by TheraView (Cablon Medical B.V.), installed onto a Siemens Primus linear accelerator, and integrated with a Lantis record and verify system, an Oldelft simulator with Digital Therapy Imaging (DTI) unit, and a Philips ADAC Pinnacle treatment planning system (TPS). This system integrates well with existing equipment and its software can process DICOM images from other sources. The system provides a complete imaging system that eliminates the need for separate software for portal image viewing, interpretation, analysis, archiving, image guided radiation therapy and other image management applications. It can also be accessed remotely via safe VPN tunnels. TheraView EPID retrofit therefore presents an example of a less expensive alternative to linear accelerator manufacturers’ proprietary EPIDs suitable for implementation in third world countries radiation therapy departments which are often faced with limited financial resources.

## INTRODUCTION

The aim of external beam radiotherapy is to deliver a tumoricidal radiation dose while minimizing dose to the surrounding normal tissues, which requires accurate and reproducible field placement. Verifying patient position and fields historically has been accomplished via exit radiation portal films. Ideally, films should be performed every day before the treatment [1-4], but this process ties up departmental personnel, the film processor, and the treatment machine for several minutes, while the patient must remain immobile on the treatment table.

The electronic portal imaging device (EPID) [5] is a relatively new development in portal imaging. Boyer *et al* [6] and Munro [7] have written comprehensive reviews of EPIDs, and, briefly, they consist of an image acquisition unit fitted to the linear accelerator (Linac), and a component that digitizes and displays these images on a computer screen. The unit should provide high resolution and high contrast images, to allow rapid verification of treatment field shape and position immediately after the patient's X-ray exposure. Recent developments include the software to analyze portal images and compare them with treatment planning images for setup accuracy and localization.

Digital X-ray images have several advantages over X-ray films: less handling, less radiation to the patient, more convenient patient management, immediate image viewing, computer-aided analysis, and storage in digital format rather than in film stacks. EPIDs provide images with better visibility and review accuracy than do films in the megavoltage X-ray energy range [8].

The main idea behind portal imaging is to ensure the patient is in the correct position during treatment, and regular portal imaging protocols reduce the size and frequency of field placement errors. Computer algorithms for detecting field displacements are better than manual approaches [9]. This process requires a reference image which shows the patient in the correct position, and the treatment portal image(s) for comparison. The reference image may be a simulator X-ray, another portal image, a digitally reconstructed radiograph or a digitally reconstructed portal image. Making image registration a routine process in clinical practice requires an integrated system that combines the functions of preparation of the reference image, portal image field edge detection, field edge matching, anatomy matching and presentation of results [10]. Another important application for portal imaging is to verify beam collimation and block/MLC shape.

The global definition of Image Guided Radiation Therapy (IGRT) involves the entire position verification process from portal image acquisition to image registration by aid of computer software. The process requires the importation of the DICOM coordinate system from the CT or virtual simulation, recording of the prescribed coordinate offsets from the simulation origin. After the images have been acquired, the position offsets are calculated instantly to provide the necessary treatment couch shifts information for re-positioning of the treatment area. After re-positioning, the re-verification images may be taken again and the circle is repeated according to departmental protocol. Consequently, an ideal IGRT system should be capable of importing the set up coordinates, recording of current target localization offsets at the time of image acquisition and thereafter the application of the total offsets to the treatment couch position.

There are a wide range of problems [11-12] that third world countries face in radiation therapy and imaging facilities. The clinical relevance of portal imaging technology in developing countries constitute a subset of these problems and faces stiff challenges because of the unfavorable economic situation. Moreover, even the institutions that can afford to purchase these state-of-the-art technologies in developing countries may not do so because of limited knowledge of their potential clinical benefits and cost-effectiveness.

Adopting EPID technology can pose problems for small radiation therapy centers, since many operate machines manufactured before EPIDs became standard equipment, or did not purchase EPID technology on more recently acquired Linacs. Adding an EPID to an existing Linac can be challenging for several reasons.

Firstly, the cost of a Linac manufacturer's proprietary add-on EPID can represent more than half the cost of a new Linac (see Table 1 for more details). Secondly, there can be hardware and software compatibility issues with third party products. Thirdly, most add-on EPID systems do not come with complete surrogate [13] based- image guided radiation therapy (IGRT) portal image management software. Finally, there can be logistic issues with the new EPID retrofit and existing Linac service maintenance contracts due to any required modifications of the Linac. In evaluating an add-on EPID system to meet portal imaging needs, to increase efficiency, and to keep the radiation therapy center competitive, it is necessary to evaluate the quality of imaging, initial purchase cost, and total cost of ownership of several systems.

**Table 1:** These are turn-key costs associated with different EPID systems that the authors evaluated as of year 2005. There was a one year warranty on all the systems. The table indicates the imaging technology used by the system, and whether the base purchase price included the image manipulation software. Prices for the different systems in this table represent quotations provided to the authors at the time the different systems were being evaluated, and may differ from current prices.

**EPIDs compatible with Siemens Primus LINAC**	**Type of Technology**	**Base price (US $) (as of the time of implementation)**	**Comment**
**OPTIVUE 500** (Siemens Medical Solutions USA, Inc, 51 Valley Stream Parkway, Malvern PA 19355)	Solid-State aSi flat panel detector	487,320.00	Comes with new Siemens LINACs. Mounted at the base of the gantry. Limited image manipulation tools. 2D and 3D viewing software optional
**BeamView TI** (Siemens Medical Solutions USA, Inc, 51 Valley Stream Parkway, Malvern PA 19355)	LCD Camera	220,660.00	Gantry mounted. Needs additional image manipulation tools. No surrogate based IGRT software included
KODAK 2000RT CR Plus system (Carestream Health Inc., Rochester, N.Y)	Film-cassette with CR film processor	200,000.00	Not mounted at the LINAC. Not “real time”. Needs additional surrogate based IGRT software
**TheraView Digital Imaging System** (Cablon Medical B.V. Klepelhoek 11, 3833 GZ Leusden, The Netherlands)	Water cooled Solid- State CCD Camera	189,900.00	Mounted at base of Linac. Price includes surrogate based IGRT software.

## REVIEW OF EPI SYSTEMS

The three major radiation therapy digital imaging technologies include camera based detectors [13-19], liquid ion chambers [20-21] and solid-state amorphous silicon detectors [22-23]. The earliest EPIDs were camera based. Here, the X-ray beam excites a metal fluorescent phosphorus screen, which converts X-rays to light, and the image is transferred to a high-resolution charge-coupled device (CCD) digital camera via a high reflectance mirror positioned at a 45-degree angle under the fluoroscopic screen. The camera control unit transforms the digital image gathered from the CCD camera via a fiberoptic-linked [24] datastream fed directly to a fiberlink personal computer interface (PCI) in the host computer to be processed by the digital image processor. This processor digitizes images, and an appropriate number of frames are averaged to reduce artifact and produce a final display image.

There are cooled and non-cooled CCD camera systems commercially available for non-radiation therapy imaging applications. The cooled CCD camera can handle very high signals per pixel, without compromising the low-level imaging performance. They are designed to allow detection of small differences in light intensity. Consequently, details of very low contrast images can be seen against much brighter backgrounds, without saturating the higher intensity areas. This capacity of detecting low contrast images, with the high sensitivity and wide dynamic range of the CCD, lead in performance indices for cooled CCD cameras that surpass the non-cooled CCD camera imaging systems [25]. Cooled CCD camera systems employ thermoelectric primary cooling with either air or water-cooled heat exchangers. The ability to remove excess heat from the heat sink depends on the method of cooling. The temperature of the cooling air or water influences the lowest operating temperature. The CCD array can be cooled to -75°C with air-cooling. Water-cooling can push the array temperature to -90°C, with an increase in the lifespan of water-cooled CCD camera compared to air cooled [21] cameras.

Liquid ion chamber arrays EPIDs have slow scan speed. As a result they have limited use in verification of dynamic techniques, such as intensity modulated radiotherapy where the dose rate can be varied during the treatment due to the variation in Linac output [26]. Because of this limited application, the authors did not pursue them further in this work.

Camera based detectors are cheaper and more durable than amorphous silicon detectors, but have relatively lower image resolution since they use phosphor screens [27] with lower light collection capability [28] to capture the image for the camera. There is also poor optical coupling between the light emitter and camera system [29]. Flat-panel amorphous silicon imagers of the same screen yield higher quality images than CCD imagers at 2-4 monitor-units (MU) exposures, but are susceptible to radiation damage to the peripheral electronics [30]. There is also need to calibrate and regularly re-calibrate these detectors for dark current and flood-field uniformity.

The lifespan of an amorphous silicon-based system can be shorter than expected with intensive IGRT imaging needs, because radiation affects the leakage current of the diodes employed in amorphous silicon detector systems, degrading the system performance [30] and accounting for the shorter lifespan compared with water-cooled camera based detector systems.

Financial calculations for the US case (based on 2008 medical billing reimbursement guidelines) for camera-based portal imaging indicate that an add-on EPID should pay for itself within two years of ownership. Additionally, the replacement cost of an amorphous silicon detector represents about 80% of the cost of a new purchase. The replacement cost for the CCD camera head would be less – about 7.5% of the cost of a new camera based system, and because of its durability, could be expected to have a service lifespan comparable to even a new Linac. In evaluating an add-on system, it is desirable to select one with a lifespan comparable to the remaining years of service of the Linac. Thus, a camera-based system is more appealing from a financial point of view.

## MATERIALS AND METHODS

### Selecting an add-on EPID

One of the categories of portal imaging devices retrofits to consider is the amorphous silicon proprietary system. The Siemens Primus aSi flat-panel proprietary system commercially available is called OPTIVUETM500 (Siemens Medical Solutions USA, Inc, 51 Valley Stream Parkway, Malvern PA 19355). This system is Linac gantry base-mounted. Its purchasing cost as an add-on EPID was greater, as seen in Table 1. Furthermore this system does not come with surrogate based IGRT software suite. Thus additional third party IGRT software would have to be purchased at an additional cost. As mentioned above, the lifespan of aSi EPID may decrease with the intensive use required to meet IGRT needs, however, over five years service are possible with less intensive usage and optimal maintenance.

The second category of add-ons is the camera based portal imaging system. There are two camera based EPID systems commercially available to choose from for retrofitting on to Siemens Primus Linac. One is the Siemens Primus Linac-specific LCD camera-based system, which requires a purchase of additional image management software to use for surrogate based IGRT. This system known as Beamview TI Plus® (Siemens Medical Solutions USA, Inc, 51 Valley Stream Parkway, Malvern PA 19355), includes a retractable and collapsible Linac gantry head-mounted detector assembly with collision detection, which can be used at any gantry angle. Beamview TI Plus® is based on target integration technology, making it capable of acquiring portal images with a very low number of Monitor Units. The Beamview Plus® electronic portal imaging device has been evaluated against conventional radiographic films and found to provide significantly "visible" or better images [31].

The other camera based portal imaging system is a third party non-Linac-specific system called TheraView system. The TheraView system (Cablon Medical B.V. Klepelhoek 11, 3833 GZ Leusden, The Netherlands) is Linac gantry base-mounted with collision detection. It is a water cooled CCD camera system and comes with a complete software imaging suite for surrogate based IGRT image review and manipulation. Its potential use for *in vivo* dosimetric applications have been explored recently [32].

The third category of the portal imaging to consider is a luminescence based system. There was only one system in this category that was commercially available at the time of the implementation of this project. This is not a retrofit system since it does not require mounting on the Linac. This system, the Kodak 2000RT CR Plus system (Eastman Kodak Comp., Rochester, NY, USA) uses EC-L cassettes with metal phosphor plates for luminescence radiography. It is a mobile system that requires no mounting. After portal irradiation, the EC-L cassette with metal phosphor plates would be physically transferred by a therapist to a computed radiography (CR) digitizer where laser scanning of the irradiated phosphor causes luminescence, whose detection is used to form a digital image [33]. The images are then electronically submitted to a computer for review. Additional software at an additional cost from another third party vendor is required to perform surrogate based IGRT image review and manipulation.

Unlike conventional radiographic film, the metal phosphor plate used with this imaging system is reusable and no film processor is required; the quality of digital images from this system is comparable to conventional film images [31], but unlike amorphous silicon or camera-based systems, the imaging is not "real time," since cassettes must physically be manipulated before images are available for review.

In all the three categories of EPID retrofits considered, the TheraView system was the most economical of all the four systems (refer to Table 1 for details on different types of EPIDs).

### TheraView portal imaging system

**Figure 1:**
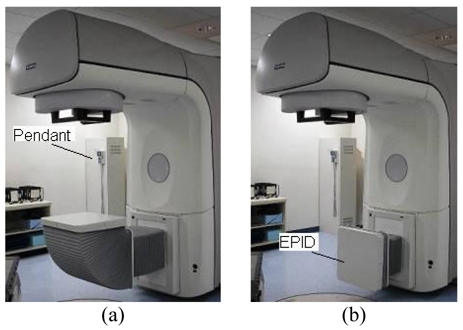
TheraView EPID unit installed at the base of Siemens Primus Linac. (a) The EPID camera unit is in position to acquire images. (b) TheraView EPID unit installed at the base of Siemens Primus Linac. EPID camera unit is in retracted position.

The TheraView Imaging System is manufactured in the Netherlands. Since Dutch power ratings differ from those in the US, factory engineers must verify that the system's power rating meets US specifications before importation. The system consists of a water-cooled, telescoping, motorized image detector with a CCD digital camera. It has a scintillator screen size of 40 cm by 40 cm with a resolution of about 0.78 mm. The camera has 1024 by 1024, 12-bit resolution with a 28 cm by 28 cm field of view. The hardware mounts easily at the base of the Siemens Primus gantry (See Figures 1a & b), at the same location where the proprietary Siemens Primus EPID is usually mounted. The pendant cord passes through the existing conduit to the modulator and is easily mounted as seen in Figures 1a and 1b. The small remote hand pendant can be mounted anywhere within the treatment room. The camera unit weighs about 80 kg, which is less than the weight of proprietary aSi EPID, and there is no interference with the existing Siemens Primus Linac. (See Table 2 for technical specifications of EPID systems considered). The Theraview EPID image characteristics have been described before [8].

**Table 2:** Technical specifications of different types of portal imaging technologies.

**Technical specifications**	**Solid-State aSi flat panel detector**	**LCD Camera**	**Digital luminescence radiography**	**Water cooled Solid-State CCD Camera**
	**Siemens OPTIVUE 500**	**Siemens Beamview TI**	**KODAK 2000RT CR Plus System**	**TheraView Digital Imaging System**
FOV at detector level	41×41 cm	40×30 cm	35×43 cm	40×40 cm
FOV at Isocenter	29×29 cm	32×27 cm	NA	28×28 cm
Spatial resolution mm/pixel	0.4	0.8	0.255	0.78
Pixel format	1024×1024 12-bit Grayscale	512×512 12-bit Grayscale	1200×1600 10-bit Grayscale	1024×1024 12-bit Grayscale
Readout (max) frames/second	3.5	3.0	NA	3.5
MTF (F50) 6MV	0.41	0.2	NA	0.34
Weight (kg)	96.9			80
Mounting location	Gantry base	Gantry head	Portable (under treatment table)	Gantry base

### Installation process

The TheraView hardware installation process took approximately 1.5 days over a weekend. The team comprised four individuals - two factory-based engineers and two local technicians, one familiar with the Siemens Primus Linac and the other being the local TheraView systems imaging engineer who would provide the maintenance services. In addition, after the system was connected to the local area network, the software engineers in the Netherlands were able to offer remote trouble shooting and guidance services to the team through the VPN tunnel, which avoided the need for local IT personnel. The software can be customized to users’ needs at any time without having to wait for the release of a newer version.

### Networking and integration with the existing equipment

The DICOM-compatible TheraView portal imaging system was networked on the existing intranet. The Oldelft simulator has Digital Therapy Imaging (DTI), a digital image acquisition, processing, and review system for image intensifier-based X-ray procedures adapted for use with a Simulix-HP simulator. The images can be stored on a hard disc with the Simulix positions and patient data, and can be retrieved for review, digital imaging processing, text and line annotation, printing and transmission to the department network or PACS. The DTI unit was configured to communicate with the TheraView system.

**Figure 2:**
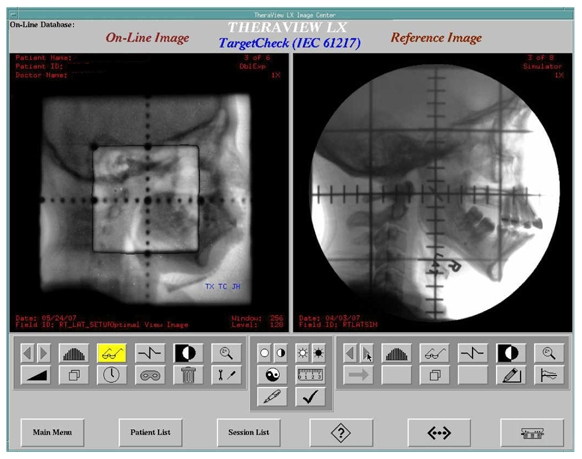
TheraView imaging system software. EPID (left) and conventional DTI (right) images for a head and neck treatment as shown. Bony landmarks were used for field matching.

The TheraView system connects to DTI and imports the DICOM images. These images can then be used as reference images for comparison with the EPID images during the initial treatment verification (see Figure 2) using the TheraView system.

The TheraView system can accept beam’s eye view field shapes as bitmap or DICOM images from the Philips Pinnacle ADAC treatment planning system. This involves configuring the TheraView system to receive images from the treatment planning system (TPS).

### Portal image processing

**Figure 3:**
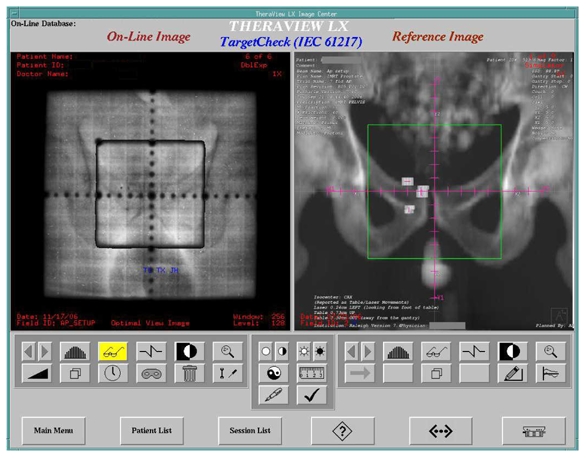
TheraView imaging system software. EPID (left) and DRR (right) images with gold markers implanted into the prostate are shown.

**Figure 4:**
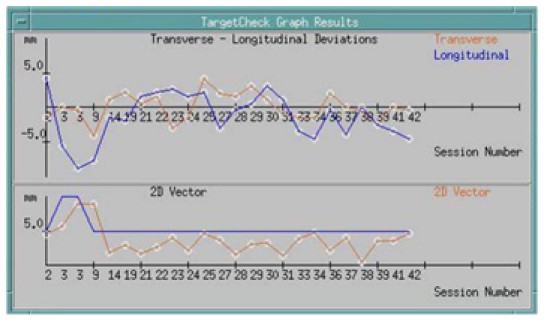
Graphic display of set up deviations over the course of 42 IGRT treatments for a head and neck cancer patient.

Manipulation of EPID images beyond the screen display requires additional software in many other EPID systems. Many cancer centers utilize record and verify systems, like IMPAC or Lantis, for the physicians to review and approve the portal images. Vendors provide non-numerical image management modules associated with their existing record and verify systems at an additional cost, as high as 30% of the cost of an add-on EPID equipped with image management tools. Within the TheraView system, there is a software component (called TargetCheck) used to numerically check and compare the on-line beam’s eye view fields with the chosen reference image (either a DRR or a simulation image) [34] and to automatically analyze the field shapes and the position of the patient based upon anatomic landmarks or implanted fiducial markers [35-37]. Figure 2 compares an image acquired using the EPID (left) and a simulator DTI image (right). Figure 3 compares an image acquired using the EPID (left) and a DRR (right). The system then reports the deviations and the shifts instructions required to match the EPID image to the reference image, based on the tolerances preset by the physician and physicist. The deviations over the course of treatments can then be displayed (shown in Figure 4) or printed as numerical values for the patient's record. The software allows graphic display of the deviations with time/fractions. Thus, even in the absence of the physician, treatment can proceed provided shifts have been made by the therapist to satisfy the pre-set tolerances.

### Remote image manipulation through the internet and system maintenance via VPN tunnel

Authorized personnel can access the database remotely via the internet through a VPN tunnel, which allows remote trouble-shooting of system troubles, and allows physicians to review portal images from any computer terminal with internet connectivity. It is important to remember that computers used for remote diagnostics or physician image review that are outside the intranet and have static IP addresses pose security issues, and care must be taken to ensure that the port is closed immediately after use, so that the port is open only when in use by authorized individuals.

## DISCUSSION

The authors have installed a less expensive third party EPID onto an existing Siemens Primus Linac. This system can also be installed on Linacs manufactured by different companies such as Varian and Elekta. The TheraView electronic portal imaging system integrates well with existing equipment. It offers a complete imaging system that eliminates the need for separate software for portal image viewing, interpretation, analysis, archiving, and other image management applications. It runs on a Linux platform, and uses DICOM capability to import treatment planning images, simulation images and scanned film images for comparison with electronically acquired portal images. It has secure internet access through a VPN tunnel that allows remote trouble shooting by factory personnel as well as remote portal image review by the physician.

The major drawback with this system is with the image quality which is relatively poor compared to other non-camera based EPIDs due to loss of image resolution inherent in the CCD technology such as afterglow of previous images in the current image, "dead" pixels and specifically for Linac systems problems synchronizing with gun pulses and the so-called "shutter" effect.

A comprehensive research work is in progress to determine the proper commissioning and QA procedures especially for Theraview system to come up with appropriate consistent parameters for MTF, spatial resolution, contrast resolution, signal to noise, typical exposure settings, positional accuracy and reproducibility, effect of the installation on machine isocentricity.

There are ongoing efforts to improve the quality of images in this system. Additionally, dosimetric applications such as measuring the entrance and exit patient doses are currently being tested as are software modules for filmless machine quality assurance, like isocenter and radiation/light field match.
